# Complete Genome Sequences of Mycobacterium Phages Tripl3t and Zeuska

**DOI:** 10.1128/MRA.00558-21

**Published:** 2021-08-12

**Authors:** Faith Cox, Tiffany Lujan, Matthew Bristerpostma, Rheaven Sandoval, Haze Murphy, Leah Dowell, Jaime Merrill, Julie Edwards, Dustin Edwards

**Affiliations:** a Department of Biological Sciences, Tarleton State University, Stephenville, Texas, USA; DOE Joint Genome Institute

## Abstract

Tripl3t and Zeuska are siphoviral bacteriophages that were isolated from Mycobacterium smegmatis mc^2^ 155 and contain double-stranded DNA genomes 53,565 bp and 53,598 bp in length, respectively. Tripl3t and Zeuska were annotated by students at Bluff Dale High School (Bluff Dale, TX) and Tolar High School (Tolar, TX) in community engagement with Tarleton State University.

## ANNOUNCEMENT

**FIG 1:**
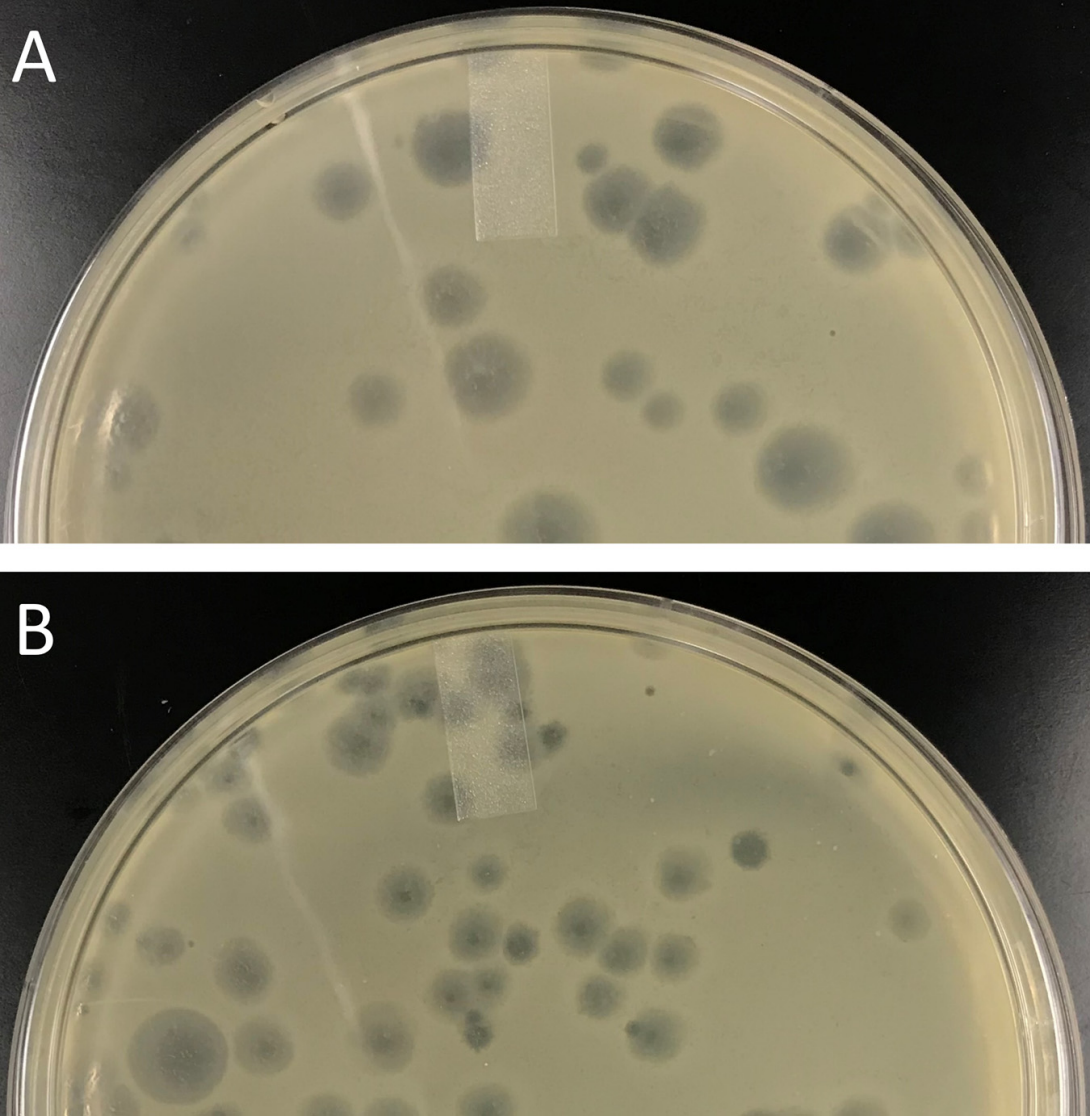
Images of plaques from bacteriophages Tripl3t (A) and Zeuska (B) incubated with Mycobacterium smegmatis mc^2^ 155 on Luria agar plates. After 24 h of incubation at 37°C, Tripl3t formed mostly large bullseye plaques and Zeuska formed medium plaques with cloudy borders.

Mycobacteriophages from the Howards Hughes Medical Institute Science Education Alliance-Phage Hunters Advancing Genomics and Evolutionary Science (SEA-PHAGES) library have been used previously to successfully treat Mycobacterium infections (1, 2). As part of the SEA-PHAGES program, we report the genomes of *Siphoviridae* mycobacteriophages Tripl3t and Zeuska, which were isolated from soil samples from Washington, DC (38.92426N, 77.01955W), and Providence, Rhode Island (41.824047N, 71.403114W), respectively. Mycobacteriophage Tripl3t was originally isolated by Demi I. F. Lewis at Howard University (Washington, DC), and mycobacteriophage Zeuska was originally isolated by Emma Herold at Brown University (Providence, RI). The samples were incubated in 7H9 liquid medium at 37°C for 2 h before the supernatant was centrifuged, filtered through a 0.22-μm filter, and incubated with Mycobacterium smegmatis mc^2^ 155 at 37°C on Luria agar plates. Bacteriophages were isolated by two rounds of picking a single, well-separated plaque, followed by dilution of samples in a 10-fold dilution series and plating with M. smegmatis mc^2^ 155 (3). Tripl3t formed large bullseye plaques, while Zeuska formed medium plaques with cloudy borders (Fig. 1). High-titer lysates were obtained by flooding “webbed” plates, as described in the Phage Discovery Guide, and DNA was extracted using the Promega Wizard DNA clean-up system (3). High-titer lysates (with dimethyl sulfoxide at a final concentration of 6.7%) were shipped on dry ice to and from the Pittsburgh Bacteriophage Institute and stored at −80°C. The Tripl3t genomic library was prepared by Virginia Commonwealth University Nucleic Acids Research Facilities using the 454 DNA library preparation kit and was sequenced with a Roche 454 GS FLX sequencer to approximately 795-fold coverage from 104,617 total reads (average read length, 165 bp). The Zeuska genomic library was prepared using the TruSeq DNA nanokit and was sequenced with an Illumina MiSeq system at the Pittsburgh Bacteriophage Institute to approximately 3,844-fold coverage from 749,005 total reads (average read length, 150 bp) (4). Raw reads were assembled to produce single-bacteriophage contigs using Newbler v2.7, and Consed v22.0 was used to check for completeness, accuracy, and genome termini (4, 5). Tripl3t and Zeuska have linear double-stranded genomes of 53,565 bp and 53,598 bp, respectively. Both bacteriophage genomes have a G+C content of 63.7% and a 10-base 3′ sticky overhang with the sequence 5′-CGGATGGTAA-3′.

Whole-genome nucleotide alignments by BLASTn (https://blast.ncbi.nlm.nih.gov) showed Tripl3t and Zeuska to have close nucleotide identity to subcluster A1 bacteriophages Wheeler (GenBank accession number NC_022070) and Abrogate (GenBank accession number KM597531) (6–8). Initial autoannotations were generated using Glimmer v3.02 (9) and GeneMark v2.5p (10), with manual revisions using DNA Master v5.23.2 (http://phagesdb.org/DNAMaster) and PECAAN (https://pecaan.kbrinsgd.org). No tRNA genes were detected by ARAGORN v1.2.38 (11) or tRNAscan-SE v2.0 (12). Putative gene functions were assigned with HHpred v3.0beta (13, 14) and BLASTp (8). All tools were run with default parameters. Putative functions were assigned to 36 of 91 predicted protein-coding genes for Tripl3t and 35 of 95 predicted protein-coding genes for Zeuska. Both bacteriophage genomes contain genes for virion assembly and structure, lysis proteins, host integration and excision proteins, DNA primase, RusA-like resolvase, RtcB-like ligase, integrase, and an immunity repressor.

### Data availability.

GenBank and SRA accession numbers are as follows: Tripl3t, GenBank accession number MK524499 and SRA accession number SRX4721441; Zeuska, GenBank accession number MK524506 and SRA accession number SRX4721438.

## References

[1] Jordan TC, Burnett SH, Carson S, Caruso SM, Clase K, DeJong RJ, Dennehy JJ, Denver DR, Dunbar D, Elgin SCR, Findley AM, Gissendanner CR, Golebiewska UP, Guild N, Hartzog GA, Grillo WH, Hollowell GP, Hughes LE, Johnson A, King RA, Lewis LO, Li W, Rosenzweig F, Rubin MR, Saha MS, Sandoz J, Shaffer CD, Taylor B, Temple L, Vazquez E, Ware VC, Barker LP, Bradley KW, Jacobs-Sera D, Pope WH, Russell DA, Cresawn SG, Lopatto D, Bailey CP, Hatfull GF. 2014. A broadly implementable research course in phage discovery and genomics for first-year undergraduate students. mBio 5:e01051-13. doi:10.1128/mBio.01051-13. 24496795 PMC3950523

[2] Dedrick RM, Guerrero-Bustamante CA, Garlena RA, Russell DA, Ford K, Harris K, Gilmour KC, Soothill J, Jacobs-Sera D, Schooley RT, Hatfull GF, Spencer H. 2019. Engineered bacteriophages for treatment of a patient with a disseminated drug-resistant *Mycobacterium abscessus*. Nat Med 25:730–733. doi:10.1038/s41591-019-0437-z. 31068712 PMC6557439

[3] Poxleitner M, Pope W, Jacobs-Sera D, Sivanathan V, Hatfull G. 2018. Phage discovery guide. Howard Hughes Medical Institute, Chevy Chase, MD.

[4] Russell DA. 2018. Sequencing, assembling, and finishing complete bacteriophage genomes. Methods Mol Biol 1681:109–125. doi:10.1007/978-1-4939-7343-9_9. 29134591

[5] Gordon D, Green P. 2013. Consed: a graphical editor for next-generation sequencing. Bioinformatics 29:2936–2937. doi:10.1093/bioinformatics/btt515. 23995391 PMC3810858

[6] Hatfull GF, Jacobs-Sera D, Lawrence JG, Pope WH, Russell DA, Ko C-C, Weber RJ, Patel MC, Germane KL, Edgar RH, Hoyte NN, Bowman CA, Tantoco AT, Paladin EC, Myers MS, Smith AL, Grace MS, Pham TT, O’Brien MB, Vogelsberger AM, Hryckowian AJ, Wynalek JL, Donis-Keller H, Bogel MW, Peebles CL, Cresawn SG, Hendrix RW. 2010. Comparative genomic analysis of 60 mycobacteriophage genomes: genome clustering, gene acquisition, and gene size. J Mol Biol 397:119–143. doi:10.1016/j.jmb.2010.01.011. 20064525 PMC2830324

[7] Pope WH, Bowman CA, Russell DA, Jacobs-Sera D, Asai DJ, Cresawn SG, Jacobs WR, Hendrix RW, Lawrence JG, Hatfull GF, Science Education Alliance Phage Hunters Advancing Genomics and Evolutionary Science, Phage Hunters Integrating Research and Education, Mycobacterial Genetics Course. 2015. Whole genome comparison of a large collection of mycobacteriophages reveals a continuum of phage genetic diversity. Elife 4:e06416. doi:10.7554/eLife.06416. 25919952 PMC4408529

[8] Altschul SF, Gish W, Miller W, Myers EW, Lipman DJ. 1990. Basic local alignment search tool. J Mol Biol 215:403–410. doi:10.1016/S0022-2836(05)80360-2. 2231712

[9] Salzberg SL, Delcher AL, Kasif S, White O. 1998. Microbial gene identification using interpolated Markov models. Nucleic Acids Res 26:544–548. doi:10.1093/nar/26.2.544. 9421513 PMC147303

[10] Borodovsky M, Mills R, Besemer J, Lomsadze A. 2003. Prokaryotic gene prediction using GeneMark and GeneMark.hmm. Curr Protoc Bioinformatics Chapter 4:Unit4.5. doi:10.1002/0471250953.bi0405s01. 18428700

[11] Laslett D, Canback B. 2004. ARAGORN, a program to detect tRNA genes and tmRNA genes in nucleotide sequences. Nucleic Acids Res 32:11–16. doi:10.1093/nar/gkh152. 14704338 PMC373265

[12] Lowe TM, Chan PP. 2016. tRNAscan-SE On-line: integrating search and context for analysis of transfer RNA genes. Nucleic Acids Res 44:W54–W57. doi:10.1093/nar/gkw413. 27174935 PMC4987944

[13] Zimmermann L, Stephens A, Nam S-Z, Rau D, Kübler J, Lozajic M, Gabler F, Söding J, Lupas AN, Alva V. 2018. A completely reimplemented MPI Bioinformatics toolkit with a new HHpred server at its core. J Mol Biol 430:2237–2243. doi:10.1016/j.jmb.2017.12.007. 29258817

[14] Söding J, Biegert A, Lupas AN. 2005. The HHpred interactive server for protein homology detection and structure prediction. Nucleic Acids Res 33:W244–W248. doi:10.1093/nar/gki408. 15980461 PMC1160169

